# Impact of self‐monitoring of salt intake by salt meter in hypertensive patients: A randomized controlled trial (SMAL‐SALT)

**DOI:** 10.1111/jch.14344

**Published:** 2021-09-12

**Authors:** Sirichai Wiriyatanakorn, Anucha Mukdadilok, Surasak Kantachuvesiri, Chusana Mekhora, Teerapat Yingchoncharoen

**Affiliations:** ^1^ Division of Cardiology Department of Internal Medicine Faculty of Medicine Ramathibodi Hospital Mahidol University Thailand; ^2^ Division of Nephrology Department of Internal Medicine Faculty of Medicine Ramathibodi Hospital Mahidol University Thailand; ^3^ Institute of Food Research and Product Development Kasetsart University Thailand

**Keywords:** hypertension, salt meter, salt, uncontrolled

## Abstract

Salt intake over reference level would result in elevated blood pressure (BP) and long‐term morbidity. Salt meter is a device used to detect sodium content in daily food. This study aimed to evaluate the efficacy of salt‐meter addition to dietary education. The authors conducted a randomized‐controlled trial in hypertensive patients with uncontrolled BP (systolic BP ≥140 mmHg or diastolic BP ≥90 mmHg). Patients were randomized to receive salt meter plus dietary education (group A) or education only (group B), and followed up for 8 weeks. The primary endpoint was change in 24‐h urinary sodium excretion. Changes in BP, salt taste sensitivity, cardio‐ankle vascular index (CAVI) were also analyzed. There were total number of 90 patients who had complete follow‐up, 45 in each group. Mean age was 62.9 years and 53% were females. Mean baseline 24‐h urine sodium was 151.6 mmol/24 h and mean SBP and DBP were 152.8 and 83.4 mmHg, respectively. Baseline characteristics were similar between two groups. At 8 weeks, mean change in urine sodium were –31.83 mmol/24 h and 0.36 mmol/24 h in group A and group B, respectively (*p* = .006). Mean decrease in BP were SBP, 14.44 versus 8.22 mmHg (*p* = .030), and DBP 5.53 versus 1.93 mmHg (*p* = .032). The salt sensitivity was improved more in group A. There was no different between change in CAVI. From this study, salt meter in conjunction with dietary education, for self‐monitoring of salt intake is superior to education alone in hypertensive patients, and provided better blood pressure control. Salt meter should be considered in uncontrolled hypertensive patients.

## 1 INTRODUCTION

Hypertension is one of the most common chronic medical conditions in clinical practice. According to Health Data Center of Ministry of Public Health, the incidence of newly diagnosed hypertensive patients in Thailand was increasing each year, from 916.89 per 100000 populations in 2014 to 1370.25 per 100000 populations in 2018.^1^ However, there was only 30% of patients whose blood pressure was well controlled.^2^ Hypertension causes multiple cardiovascular sequelae, for example, coronary artery disease (CAD), heart failure, cerebrovascular accident (CVA), and renal failure. Data from the study of Prospective Studies Collaboration group showed that every 20 mmHg of systolic blood pressure (SBP) or 10 mmHg of diastolic blood pressure (DBP) increment were associated with approximately two times risk of mortality from acute myocardial infarction (MI) and stroke.^3^ These consequences impact both public health and economy systems.

High dietary salt intake was believed to be associated with hypertension and was demonstrated in many clinical studies. Each 1‐gram (g) sodium intake over the reference level would result in 2.11 mmHg increment in SBP and 0.78 mmHg increment in DBP.^4^ In the same way as many studies about salt ingestion and blood pressure in both Western and Asian countries, the decrease in salt intake measured by 24‐h urinary sodium excretion was associated with decrease in blood pressure (up to 5.8 mmHg of SBP^5^, ^6^, ^7^, ^8^) and more pronounced in hypertensive than normotensive populations.^9^, ^10^ Furthermore, salt intake over 5 g/day or sodium intake over 2.3 g/day is associated with cardiovascular mortality as mentioned in many studies.^11^, ^12^

Dietary salt reduction campaigns have been launched in several countries; however, most of them, especially in low‐ and middle‐income countries,^13^ were unsuccessful, in part, due to time limitation in the clinic, lacking of awareness, and the higher threshold to detect salt taste in people with chronic high salt ingestion. From recent national survey, Thai people had consumed more than 9.1 g of salt per day, which was nearly two times above WHO reference level (5 g/day).^14^ Sources of excessive salt intake mostly from seasoning, preservative food, and sauce; such as fish sauce, fermented soy sauce, soybean paste, powder bouillon, and pickled fish. Most of all (more than 60%) are in the form of liquid or solution such as curry, soup, or broth. Thus, reduction in consuming seasoning, sauce, or soup would facilitate salt lowering habit.^15^

People who regularly consume high sodium diet are less sensitive to salty tasting and have higher salt taste threshold. This factor, in turn, results in the higher sodium intake afterwards.^16^ In normal population, the usual salt taste detection and recognition thresholds are 5 and 15 mmol/L of NaCl solution.^17^, ^18^ These thresholds are significantly greater in patients with hypertension, which are 15 mmol/L for detection threshold and 27 mmol/L for recognition threshold.^19^

Salt meter is a device used to measure the sodium chloride content in any particular food and display the results to guide users. Salt meter, in conjunction with comprehensive health education would pave the way to the successful outcome, which could lead to better blood pressure control and lessen the risk of cardiovascular complications.

This study aimed to evaluate the efficacy of self‐monitoring of salt intake by salt meter device in patients with uncontrolled hypertension by measuring changes in 24‐h urine sodium excretion which reflects the daily salt intake, blood pressure (systolic and diastolic BP), salt taste sensitivity, and estimating the vascular risk by cardio‐ankle vascular index^20^, ^21^ (CAVI).

## 2 METHODS

### 2.1 Study design

SMAL‐SALT study is a single‐center, randomized, controlled open‐labeled study with 8‐week treatment outcome. The study protocol had been reviewed and approved by the Ethical Committee of Human Rights Related to Research Involving Human Subjects, Faculty of Medicine Ramathibodi Hospital, Mahidol University (ethical committee [EC] approval number 04‐60‐21) and our trial was conducted in accordance with the principles of good clinical practice and the Declaration of Helsinki. This trial was registered at Clinicaltrials.gov, number NCT04286802.

### 2.2 Patient enrollment

Enrolled patients were at least 18 years of age and were diagnosed with hypertension for more than 3 months including either patients with antihypertensive naïve or currently on antihypertensive drug(s). The patients were eligible for study entry if the 24‐h urine sodium was more than or equal to 90 mmol per day (comparable to sodium intake over 2 g per day), average systolic blood pressure between 140 and 180 mmHg or diastolic blood pressure between 90 and 110 mmHg using last three consecutive hospital visits, no recent adjustment of antihypertensive agents (within 1 month), estimated glomerular filtration rate (eGFR) above 45 ml/min/1.73m^2^, and able to collect 24‐h urine.

The exclusion criteria were chronic kidney disease stage 3B and below (eGFR less than 45 ml/min/1.73 m^2^), hyponatremia (serum sodium less than 135 milli‐equivalent per liter [mEq/L]), unable to collect 24‐h urine, or denied to enter the study. Adjustment of any antihypertensive agents during study period was not allowed to minimize the confounding to the measured outcome. Patients were excluded during the study if they had systolic blood pressure over 180 mmHg, had obligatory indication to adjust antihypertensive drug regimen, or presented with hypertensive emergency such as acute myocardial infarction, heart failure, or cerebrovascular event.

### 2.3 Study objectives

The primary objective was to compare the change in 24‐h urine sodium excretion (which represents daily consumption of sodium) from baseline between group of uncontrolled hypertensive patients using salt meter for self‐monitoring of salt intake plus dietary education and those receiving dietary education alone. The secondary objectives were to compare changes in systolic and diastolic blood pressure, salt taste detection threshold (salt taste sensitivity), and cardio‐ankle vascular index (CAVI). Patient‐reported adverse events, significant electrolyte abnormality, and hypertension‐related emergency condition were also monitored.

### 2.4 Trial conduction

The study was conducted at Ramathibodi Hospital (Bangkok, Thailand) during June 2017 to January 2020. Participants were recruited from outpatient department (family medicine, internal medicine, and cardiology clinics) at Ramathibodi Hospital, Mahidol university (Bangkok, Thailand). All participants were evaluated for eligibility before randomization and follow‐up. All patients gave written informed consent before initiation of the protocol.

Hypertensive patients whose blood pressure was uncontrolled were 1:1 randomly assigned, by computer‐generated random numbers, to receive salt meter device for self‐monitoring of salt intake in addition to dietary education by dietician (group A or device group) or receive only dietary education by dietician (group B or control group).

**FIGURE 1:**
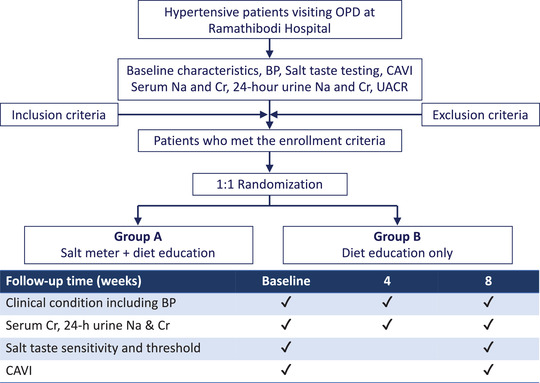
Protocol flow chart

Each patient was arranged to follow up at 4 and 8 weeks after recruitment and randomization. Patients received brief clinical interview and focused physical examination, measured weight and blood pressure, taken blood for serum sodium, creatinine and eGFR, collected urine for spot urine protein and creatinine at baseline and at 8‐week visits. Twenty four hour urine was collected within a day before placing specimen to a laboratory unit to measure 24‐h urine sodium excretion at baseline, 4, and 8 weeks (Figure 1). We estimated glomerular filtration rate (GFR) from serum creatinine based on the Chronic Kidney Disease Epidemiology Collaboration (CKD‐EPI) equation.^22^ Diary form to record use of salt meter was given to each patient in group A to evaluate the adherence and frequency of using device. The blood pressure measurement was done under instruction by the nurse and participants: avoid alcohol, caffeine, and smoking at the day of visit; taking morning antihypertensive drugs 2 h prior to measurement, emptying bladder, sitting for 5–10 min before measurement on a comfortable chair with arm support, and avoid talking while measure the blood pressure. The blood pressure measurement was done three times within 15 min and averaged using a digitalized arm‐cuffed blood pressure monitor at the separate unit from usual outpatient clinic to provide noiseless environment. This blood pressure device was calibrated and applied to all of the participants.

#### 2.4.1 24‐h urinary sodium collection

Urine samples were collected on the day of completion for determination of urine volume, sodium and creatinine excretion. Urinary sodium was determined using the indirect ion‐selective electrode method and the enzymatic method for urine creatinine assay. Urine samples were considered incomplete and discarded if (a) total urine volume was less than 500 ml in 24‐h collection; (b) estimated daily urinary creatinine excretion was less than 0.98 g/day for males and less than 0.72 g/day for females^23^; or (c) reported duration of collection was less than 24 h.

#### 2.4.2 Salt meter devices

**FIGURE 2:**
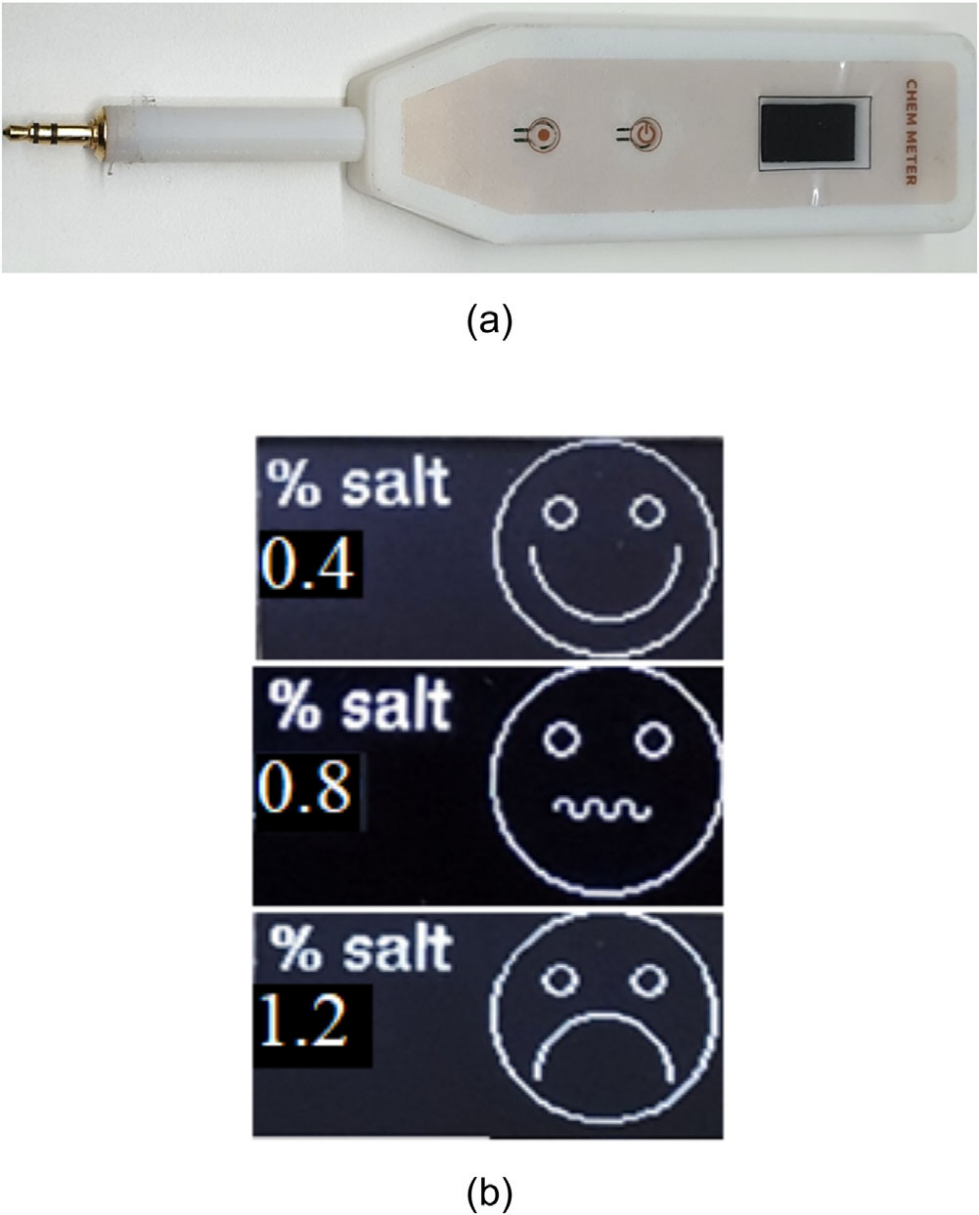
(A) Salt meter devices used in this study (developed in collaboration with Faculty of Engineering, Mahidol University, Nakorn Pathom, Thailand). (B) Examples of display from salt meter, the measured concentration of sodium chloride (NaCl) content in the interested food showed as percentage and emoticon facial expression

We, in collaboration with Faculty of Engineering, Mahidol University, Thailand, developed the user‐friendly version of salt meter devices using PPM/TDS meter with total dissolved solids expressed in parts per million (ppm) by measuring the electrical conductivity of sodium chloride in food and converting into ppm unit. The devices use the alternating electrical current, which has high frequency, to accurately determine the concentration of sodium chloride from a range of 0–5%. The display screen was developed to display the result in emotion or emotional face graphic that would be easy to understand for all users (Figure 2A). When the sodium chloride content is in between < 0.7%, 0.7–0.9%, and > 0.9%, there will be smile, poker face, and frown, display respectively (Figure 2B).

Validation of the salt meter could be performed by self‐calibration before each use by study participants. By applying the salt meter in standard normal saline solution and pressing the calibration button, it will automatically be calibrated to read at 0.9% salt. The participants were then informed how to do calibration when needed. Furthermore, we have compared results from our salt meter with the standard laboratory analysis for sodium in food using Inductively Coupled Plasma Optical Emission Spectrometer (ICP‐OES) Perkin Elmer Optima 8000, AOAC (2016) at the Institute of Food Research and Product Development, Kasetsart University, Bangkok, Thailand, which showed good correlation (R^2^  = 0.8559). We also performed device calibration at the follow‐up visit (week 4) for patients who received salt meter for self‐monitoring. After complete follow‐up, we collected the used devices to check for battery remaining and calibration with standard normal saline solution before giving them to the next patients.

The salt meter instruction was demonstrated by the independent staff to the patients allocated in the intervention group at the first visit and re‐evaluate the proper of use at the following visit. Participants who received salt meter were requested to use the salt meters as much as possible and for at least five times per week and recorded the kind of food consumed and results from salt meter as well as the decision of patients to‐eat or not‐to‐eat that food in salt meter diary forms given at initial visit.

#### 2.4.3 Dietary education

For dietary education, all the participants received the education about dietary recommendation in hypertensive patients from the same dietician. This dietician was certified with clinical nutrition program and have practiced in the field of cardiovascular disease. Dietician was not allowed to know the patient allocation. We did group education with individual assessment for each session. The same dataset and slide set were used for every group. The nutritional education program consisted of general healthy food consideration and focused on food and condiments that contain sodium which should be reduced or avoided. We also provided both photographs and narration on the slides. Each session took about 20 min for lecturing and 10–15 min for question and answer. The participants were assessed by each visit and re‐educate to ensure the maintenance of knowledge.

#### 2.4.4 Salt taste sensitivity testing

The salt taste sensitivity was defined as detection and recognition threshold.^19^, ^24^ We conducted the saltiness sensitivity analysis with the 3‐alternative forced‐choice (3‐AFC) technique.^25^ This technique is a simplified method and widely recommended for identifying differences in taste sensitivity among different populations as well as for studying how disease conditions affect taste sensitivity. This method is fast and prevents fatigue of participants during the test. The validity of this method has been determined.^19^

Salt solutions were prepared freshly on the day of test. On the day of the test, the participants were asked to avoid smoking, drinking any beverage, or chewing gum for at least 2 h before the tests. Additionally, to assess the effect of repeated tests, we also measured the detection and recognition thresholds in 10 healthy volunteers after a 1‐week interval and the results were consistent.

At the testing room, participants received a series of 8 3‐AFC presentations. Every presentation consisted of two blank samples (distilled water) and a salt solution (2–256 mM in 2‐fold steps). For each presentation, participants were informed to taste the samples in order from left to right by putting each sample solution all at once, gargling in their mouth and spitting. Between each series of presentations, participants rinsed their mouths with distilled water. Participants were asked to indicate which of the samples contained the taste (detection threshold) and to describe the taste (recognition threshold). If a participant perceived no difference between the three samples at any presentations, they were asked to choose one (forced choice). The individual best‐estimated detection and recognition thresholds (BETs) was calculated by the geometric mean of the last missed concentration and the next higher concentration detected, considering it as the first of all subsequent concentration correctly answered. The group best‐estimated detection and recognition thresholds were calculated considering the geometric means of the individual BETs. The salt taste sensitivity testing had been done at baseline and 8 weeks after enrollment in both intervention and control groups.

#### 2.4.5 CAVI

CAVI is a non‐invasive instrument that was used to predict arterial stiffness and is comparable with other parameters of atherosclerosis. It is measured by an electrocardiogram (ECG), phonocardiogram (PCG), and pulse‐wave velocity (PWV) from the starting point of the aorta from the heart to the ankle as well as blood pressure. This index is calculated from the heart–ankle pulse wave velocity (haPWV) adjusted for blood pressure based on a stiffness parameter. In our study, we used a VaSera CAVI instrument (Fukuda Denshi Co Ltd, Tokyo, Japan). The patients were in supine position; then the ECG, PCG, blood pressure, and pulse wave velocity were monitored and CAVI was derived from preset formulation.^26^, ^27^

### 2.5 Statistical analysis

We did a primary analysis based on intention‐to‐treat populations. We calculated the sample size with the *n4studies* program version 1.4.0.^28^ Because there was no current study that demonstrates the efficacy of salt meter device, we assumed the treatment effect of this device for the sample size calculation from the study about effects of health education to the improvement of urine sodium excretion. There was an average of 20% decrement in urinary excretion of sodium^29^; thence, we used 20% improvement for control group and estimated that there would be 30% improvement in an intervention or salt meter group. The calculated sample size was 45 patients for each group, with 80% power and significant level of 0.05.

For the statistical analyses, we used the SPSS program version 18.0. Descriptive analysis was done and showed in terms of number, percentage, and mean ± standard deviation (SD). We used Chi‐square test for any independent categorical values. For continuous values, we used Student t‐test or one‐way ANOVA for any independent data. Two‐way ANOVA was used to determine interaction between independent factors for additional analysis. All tests were prespecified to the 5% significant level.

## 3 RESULTS

A total of 100 patients were enrolled, with 90 patients who met the inclusion criteria and had complete follow‐up, 45 in group A or device group (salt meter device plus dietary education) and 45 in group B or control group (dietary education only). All patients were diagnosed as primary hypertension. There was no crossover between both groups. From all patients, mean age was 62.9 years old. 49 of 90 patients (53%) were females. 11.1% were antihypertensive‐naïve, and median number of currently received antihypertensive agents was two. Mean baseline 24‐h urine sodium was 151.6 mmol/24 h and mean baseline SBP and DBP were 152.8 and 83.4 mmHg, respectively. Mean serum creatinine and eGFR were 0.86 and 84.37 ml/min/1.73 m^2^.

Demographic data and baseline characteristics for each group were showed in Table 1. Overall, demographic and baseline characteristics including age, sex, body mass index (BMI), number of antihypertensive drugs, smoking status, baseline systolic and diastolic blood pressure, body weight, 24‐h urinary sodium excretion, serum creatinine and eGFR, and serum sodium were balanced between two groups (all *p *> .05). There was no statistically different in classes of antihypertensive drugs between two groups. Mean CAVI was 8.08 in group A and 8.64 in group B without statistically significant (*p *= .103).

**TABLE 1:** Demographic data and baseline characteristics

Baseline characteristics	Group A (salt meter)	Group B (Control)	*p‐*value
Age, mean ± SD (years)	63.2 ± 11.9	62.5 ± 10.0	.754
Female sex (%)	26 (58)	23 (51)	.672
No. of antihypertensive drugs, mean	1.9	1.7	.420
Types of antihypertensive drugs			
Diuretics	13.0 %	17.4 %	.636
ACEi or ARB	82.6 %	69.6 %	.300
Calcium‐channel blocker	56.5 %	60.8 %	.765
Beta‐blocker	73.9 %	52.2 %	.127
Smoking (%)	15.7	10.5	.497
Body weight, mean (kg)	71.8	66.6	.115
Body mass index, mean ± SD (kg/m^2^)	27.9 ± 5.3	26.3 ± 5.1	.250
Blood pressure, mean ± SD (mmHg)			
Systolic	153.9 ± 10.2	151.3 ± 12.1	.308
Diastolic	83.8 ± 8.0	82.5 ± 9.8	.513
24‐h urine sodium, mean ± SD (mmol)	153.8 ± 61.7	144.2 ± 51.9	.427
Creatinine, mean ± SD (mg/dl)	0.86 ± 0.2	0.87 ± 0.3	.728
eGFR, mean ± SD (ml/min/1.73 m^2^)	84.1 ± 17.7	84.6 ± 21.2	.903
Serum sodium, mean ± SD (mEq/l)	140.2 ± 2.2	139.1 ± 7.3	.361

*Abbreviations*: ACEi, angiotensin‐converting enzyme inhibitors; ARB, angiotensin‐II receptor blockers; eGFR, estimated glomerular filtration rate.

**FIGURE 3:**
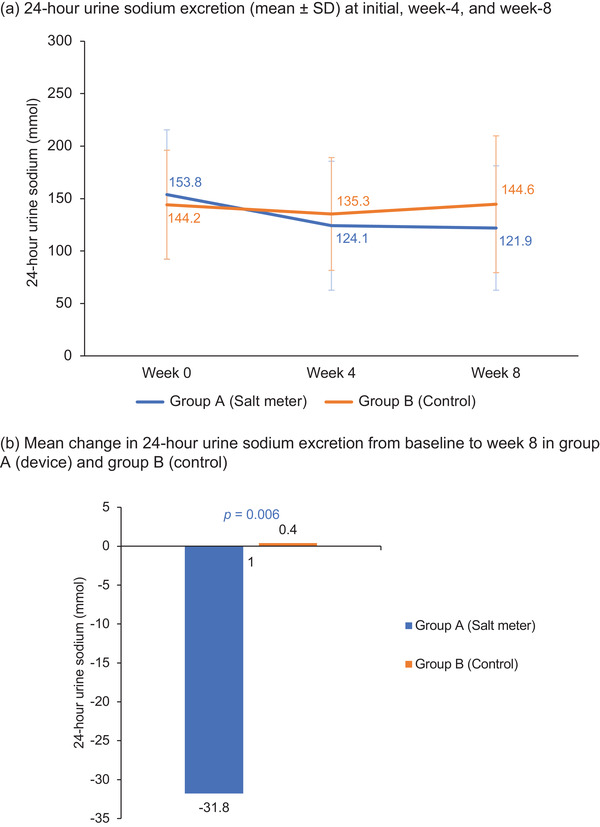
(A) 24‐h urine sodium excretion (mean ± SD) at initial, week‐4, and week‐8. (B) Mean change in 24‐h urine sodium excretion from baseline to week 8 in group. A (device) and group B (control)

Table 2 showed results of the primary and secondary endpoints at the beginning (week 0) and the end of the study (week 8). With intention‐to‐treat analysis for the primary endpoint, at week 8, mean change in 24‐h urine sodium were –31.83 ± 49.2 mmol/24 h and 0.36 ± 59.4 mmol/24 h in group A and group B, respectively (*p *= .006). This signified lower daily sodium consumption in group A (device group) compared to group B (control group) (Table 2, Figure 3). We have analyzed data by two‐way ANOVA for two independent variables including intervention‐received, stage of hypertension, medication or treatment‐naïve, and degree of initial urine sodium excretion, which showed no significant effects or interaction on primary outcome.

**TABLE 2:** Primary and secondary outcomes (mean ± SD)

	Group A (salt meter)			Group B (control)			
	Initial	Week 8	Change	Initial	Week 8	Change	*p*‐value
**Primary endpoints**							
24‐h urinary sodium excretion (mmol)	153.8 ± 61.7	121.9 ± 59.3	–31.8 ± 49.2	144.2 ± 51.9	144.6 ± 65.2	0.4 ± 59.4	.006
**Secondary endpoints**							
Blood pressure							
Systolic blood pressure (mmHg)	153.9 ± 10.2	139.5 ± 13.4	–14.4 ± 14.2	151.5 ± 12.1	143.3 ± 16.0	–8.2 ± 12.6	.030
Diastolic blood pressure (mmHg)	83.8 ± 8.0	78.2 ± 7.5	–5.5 ± 7.3	82.5 ± 9.8	80.5 ± 9.8	–1.9 ± 8.1	.032
Salt taste sensitivity							
Salt taste detection threshold	7.57 ± 0.31	6.03 ± 0.31	–1.27 ± 0.07	7.02 ± 0.39	6.75 ± 0.39	–0.27 ± 0.79	.243
Salt taste recognition threshold	13.65 ± 0.48	12.95 ± 0.34	–0.70 ± 0.10	13.17 ± 0.39	11.58 ± 0.43	–0.62 ± 0.10	.928
Cardio‐ankle vascular index (CAVI)	8.08 ± 1.21	8.21 ± 1.55	0.02 ± 1.33	8.65 ± 0.99	8.43 ± 1.11	–0.11 ± 0.83	.738

**FIGURE 4:**
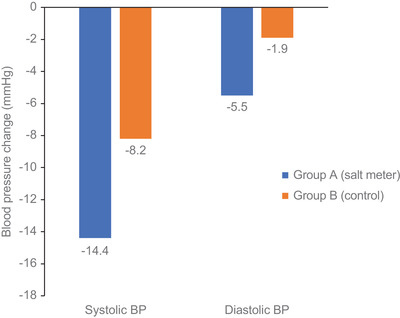
Mean change in systolic and diastolic blood pressure from baseline to week 8 in group A (device) and group B (control)

For secondary endpoints (Table 2), mean change in systolic blood pressure in each group were –14.44 ± 14.2 mmHg in group A and −8.22 ± 12.6 mmHg in group B (*p *= .030). Mean change in diastolic blood pressure was –5.53 ± 7.3 mmHg in group A and –1.93 ± 8.1 mmHg in group B (*p *= .032) (Figure 4). Furthermore, at the end of follow‐up the salt taste sensitivity evaluated by salt detection threshold was lower in group A and showed the trend toward improvement in group A (detection threshold decrement = 1.274 ± 0.072) than that of group B (decrement 0.265 ± 0.785). Though, there was no significant different between change in salt taste recognition threshold or change in CAVI between two groups (0.189 versus ‐0.108, *p* = .738). All patients have more motivation to maintain low salt diet after completion of the study. No adverse event or critical laboratory abnormality was observed throughout the study.

Concerning the adherence of salt meter device usage, all patients who received the salt meters were in good compliance, according to the salt meter diaries which they had reported to the investigators during the follow‐up period.

## 4 DISCUSSION

In uncontrolled hypertensive patients who are on antihypertensive medications or those who are treatment‐naïve, high dietary salt intake is considered to be an important contributing factor. Our study shows that, in addition to usual care and comprehensive dietary education about sodium reduction by dietician, salt meter device for self‐monitoring of salt intake has a superiority in efficacy to reduce 24‐h urine sodium excretion and blood pressure compared with dietary education alone. Reduction in 24‐h urine sodium excretion reflects the lowering in dairy sodium consumption. Additionally, there is a trend in improvement of salt detection threshold that potentially be the factor for maintaining low‐salt consumption behavior.

Based on what we know, this is the first randomized‐controlled trial focusing on the role of salt meter device as an assisted tool to the salt reduction strategy and blood pressure control in uncontrolled hypertensive patients. Diuretics use, as a possible confounding factors of urinary sodium excretion, was similar between two groups (Table 1). Our primary outcome showed significant reduction in 24‐h urine sodium excretion with an average of 31.83 mmol/day. This is comparable and rather more noticeable to previous studies on sodium reduction interventions. Li and coworker, ^30^ studied an effect of health education on community‐based adults (either normotensive or hypertensive) showed 14 mmol/day decrement in 24‐h urine sodium excretion. A randomized‐controlled trial focusing health behavior education in Chinese hypertensive patients showed 19 mmol/day reduction, similarly.^31^ Other interventions such as salt substitution^32^, ^33^ or self‐monitoring of urine electrolyte excretion^34^ had smaller effect size, approximately reduction of 5 to 8 and 13.7 mmol/day, respectively. Whereas, National Health Survey of England during 2003 to 2011^12^ which was an observational data evaluating national salt reduction program showed overall 32.2 mmol reduction in 24‐h urine sodium excretion; however, this was national survey which may have ecological bias and potential confounding factors. From our study, salt meter device used has remarkably potential to support salt reduction policy.

Regulating salt intake behavior in hypertensive patients would finally result in better blood pressure control. Subsequently, better blood pressure control would affect any future morbidity and mortality associated with hypertension. There are many evidences revealed causal relationship of quantity of salt intake and blood pressure.^35^, ^36^ Furthermore, a smaller number of antihypertensive agents is another possible benefit in term of health economics and treatment‐related adverse reactions.

Because of many possible confounding factors contributing to the blood pressure, we decided to use the less‐confounded objective outcome which is urinary sodium excretion as a primary endpoint instead of the blood pressure. With maximal effort to minimize the confounding factors, randomization showed similarity in baseline characteristics data between two groups. In term of blood pressure control, patients in group A or device group have pronounce magnitude in both systolic and diastolic blood pressure lowering; 14.44 and 5.53 mmHg, respectively. These numbers are significantly lower in those of control group with differences of 6.2 mmHg for systolic blood pressure and 3.6 mmHg for diastolic blood pressure. Despite the minimal change in 24‐h urine sodium excretion in group B, there was small decrement in blood pressure. We believed that the effect of BP lowering seen in this study was partly from Hawthorne effect. However, there would be Hawthorne effect in both groups, but more pronounced BP lowering in group A could be attributed by salt meter intervention. The effect size in our study is rather larger when compared to DASH diet which reduced systolic blood pressure by 2.8 mmHg and diastolic blood pressure by 1.1 mmHg more than control diet.^37^ From meta‐analysis of overall effects of salt reduction interventions, including health education and salt substitution, mean effect was ‐4.3 mmHg in lowering systolic blood pressure and ‐1.6 mmHg in lowering diastolic blood pressure.^35^ Moreover, when compared to antihypertensive monotherapy, results from our study show approximately half an effect to the average of angiotensin‐converting enzyme inhibitors which were about 11.5 and 8.6 mmHg lowering in systolic and diastolic blood pressure, respectively.^38^ This significant effect may be from the high salt diet consumption and salt‐sensitive in elderly patients in the study.

In hypertensive patients, the salt taste sensitivity is worse compared to healthy population^19^ and it relates with the preference and familiarity to salty taste.^39^ These are obstacles to reduce salt consumption for chronic hypertensive patients and chronic high salt‐intake people. With the use of salt meter, patients can differentiate the foods with high content of salt and avoid them. Besides, gradually decrement in amount of salt exceeds the ability of taste bud to distinguish the change. Previous evidence supported that reduction in salt intake for at least 1 week can change the threshold for salty taste.^40^ This would be the reason why patients in group A, which had more reduction in salt intake indicated by lower urinary sodium excretion, had better detection threshold than those in group B.

Conversely, this trial could not demonstrate the significant change in CAVI parameter between two groups at the end of follow‐up. This might be from the relatively short period of follow‐up to observe the vascular change. Longer duration of follow‐up may require to see the change in atherosclerotic outcome.

Somehow, there were some limitations of our study such as non‐blinded study, since the salt meter device in the intervention arm could not be blinded, neither using placebo in the control arm which might be harmful and unethical. However, we used an objective outcome measurement rather than subjective outcome as well as many approaches to reduce the bias. Dietary education was arranged in the same directions for all participants with same dataset for teaching and certificated dietician who did not know for the patients’ allocation. Blood pressure measurement and salt taste sensitivity testing were done with protocol‐directed guidelines by the staffs who did not know for the patients’ allocation.

Salt meter as a device for self‐monitoring of salt intake will facilitate hypertensive patients who have chronic high sodium consumption behavior to be more awareness, self‐learning to food with low sodium content, and will gradually improve their salt taste sensitivity. Due to technical design of salt meter that it cannot apply to the solid object or food, some food may be not suitable for testing with salt meter device. Nevertheless, we postulated that use of salt meter would habituate patients to learn which food has high or low content of sodium and subsequently results in eating habit adaptation. Eventually, people may not necessarily need the device for regular monitoring. Lowering salt intake could reduce salt‐water retention and regulate the renin‐angiotensin‐aldosterone system which finally result in blood pressure decrement.^36^

In conclusions, salt meter in conjunction with dietary education, for self‐monitoring of salt intake is superior to education alone in hypertensive patients, and provided better blood pressure control. Improvement in salt taste sensitivity will facilitate the long‐term benefit. Salt meter should be considered in patients with uncontrolled hypertension.

## CONFLICTS OF INTEREST

None declared.

### AUTHOR CONTRIBUTIONS

Sirichai Wiriyatanakorn Project administration, Study design, Acquisition of data, Analysis of data, Drafting and revision of manuscript, Anucha Mukdadilok Acquisition of data, Surasak Kantachuvesiri Study conception and design, Revision of manuscript, Chusana Mekhora Acquisition of data, Analysis of data, Teerapat Yingchoncharoen Corresponding author, Study conception and design, Funding acquisition, Revision of manuscript.
